# Prevalence of overweight and obesity among primary school children in a developing country: NW-CHILD longitudinal data of 6–9-yr-old children in South Africa

**DOI:** 10.1186/s40608-014-0030-4

**Published:** 2015-02-07

**Authors:** Anita E Pienaar

**Affiliations:** Physical Activity, Sport and Recreation (PhaSRec), Faculty of Health Science, North- West University, Private Bag X6001, Potchefstroom, 2520 Republic of South Africa

**Keywords:** Overweight, Obesity, Children, Race, Gender, SES, Developing countries

## Abstract

**Background:**

Widespread trends of increasing child obesity are reported in developing countries. This longitudinal NW-CHILD study investigated changes in overweight and obesity over a three year period among 574 children between the ages 6 and 9 (282 boys, 292 girls; 407 black, 143 white) in South Africa (SA), taking into consideration sex, race and school type. Stratified random sampling was used to identify 20 schools, across 5 school SES levels (quintiles), in 4 educational districts of the North West Province of SA. Standard anthropometric techniques and international age adjusted BMI cut-off points for children were used to determine overweight and obesity, 3-years apart. Mixed models were used to analyse the effects of sex, race and socio-economic status (SES) of the school.

**Results:**

Overall obesity increased over 3-years by 4% from 12.5% at baseline to 16.7% during follow-up. Obesity increased significantly in both white (4.2%) and black (2.0%) children, although overall prevalence in the final year was double (27.3%) in white children compared to black children (13.3%). Prevalence in obesity increased more in boys (3.2%) compared to girls (2.4%), although girls showed a higher overall prevalence (18.5%). SES effects were significant where children in schools associated with higher SES, had the highest rate of increase and the highest prevalence of obesity. A significant change towards an unhealthy BMI was found in 9.2% of the group over the 3-year period, although a small percentage (3.0%) also transitioned towards a healthier BMI.

**Conclusions:**

Overall obesity prevalence rose significantly from 6–9-years. Obesity, compared to overweight, increased more during this period. Prevalence and rate of increase differed markedly in different sexes, race and SES, masking the extent of the problem. Shifting towards an unhealthy BMI was more common than obtaining a healthier BMI over the 3-year period. It also demonstrated the difficulty of breaking the cycle of obesity, once it had started. Early prevention strategies are needed based on the trends established in this study, with special attention to white children living in high SES regions, and black children in economic transition.

## Background

The Global Burden of Disease Study [1] describes obesity as a global health challenge showing widespread increasing trends over the past decades, with no national success stories of decreasing trends over the past 33 years. The prevalence of obesity in children rose worldwide by 47.1% between 1980 and 2013 [1]. Overweight and obesity, which were previously considered problems afflicting mainly the affluent, are now markedly on the increase in low and middle income countries, particularly in urban areas [2-4]. Globalisation, improving economic conditions and changing dietary habits in developing countries are purported as responsible for the rapid increase in obesity [3]. This increase is associated with a lack of supportive policies in sectors such as health, agriculture, transport, urban planning, environment, food processing, distribution, marketing and education. Presently, it is estimated that more than 30 million overweight children live in developing countries and 10 million in developed countries [3]. Available estimates for the period between the 1980s and 1990s show that the prevalence of overweight and obesity in children increased by a magnitude of two to five times in developed countries (e.g. from 11% to over 30% in boys in Canada), and up to almost four times in developing countries (e.g. from 4% to 14% in Brazil) [5]. Globally, increasing prevalences for developing countries are reported from 1980 to 2013 in children and adolescents showing changes from 8.1% to 12.9% in boys and from 8.4% to 13.4% in girls [1]. In 2010, 43 million children (35 million in developing countries) were estimated to be overweight and obese, with 92 million at risk of overweight [6].

The fastest growth rates of obesity among pre-school children are found in Africa where the numbers of overweight and obese children in 2010 were more than double those reported in 1990 [6]. Results of a systematic review [4] further substantiate reports of overweight/obesity transition among school-aged children in Sub-Saharan Africa. Statistics furthermore indicate that South Africa (SA) has amongst the highest child obesity rates in Africa [2,4]; the prevalence of obesity among South African children is comparable to that found in developed countries more than a decade ago [7].

A review summarizing obesity among South African children, from birth to the age of 19 years, indicates low overweight and obesity rates before 1999, with more recent studies showing a mean prevalence of just over 15% for overweight and obesity combined [8]. This review reported that this prevalence does not give a true reflection of the problem, because overweight and obesity differ markedly between age groups, boys and girls, ethnic groups, and geographical areas. Findings also showed a significant increase in overweight and obesity from 1999 to 2004, based on reported prevalence’s of cross-sectional studies [7].

Only a few cross-sectional studies and trend analyses have reported on obesity prevalence in adults and children in SA [2,9]. Rates of overweight and obesity among children between 13–19 years (grades 8–11) from the first National Youth Risk Behaviour Survey in 2002 in SA showed overweight prevalence of 6.9% for boys and 24.5% for girls, and obesity prevalence of 2.2% for boys and 5.3% for girls respectively [2]. The researchers’ remark that it is difficult to determine whether the rates observed in their study represent an increase in prevalence, although their data indicate an expanding ‘epidemic’ in obesity and related chronic diseases [2]. They concluded that consistent with data from other countries in transition, it is highly likely that overweight and obesity prevalence rates today are higher than those found 10 to 20 years ago.

Temporal trends in obesity among children and adults in SA have been reported, based on a 2005 nationally representative sample of 1–9-year-old children and a sample of 16–35-year-old adolescents and adults, compared with study populations from the SA National Food Consumption Survey (NFCS) 1999. Data were re-analysed according to the WHO 2006 and 2007 reference values [9]. Taking into account the limitations of a comparison between the 1999 and 2005 national data, a significant decrease was however seen in overweight based on BMI nationally (17.1% to 14% (10% overweight and 4% obese). An overall overweight and obesity prevalence of 10.3% is also reported among 7–9 year-old children [9].

Time trend analyses of obesity prevalence, based on representative and national surveys, have also been performed in developed countries including Portugal [10], Japan [11], Slovenia [12], UK [13], USA [14,15] and Canada [16]. Some report rising obesity prevalence among child populations [10,11], although trends of levelling-off and stabilizing prevalence’s are also reported (e.g., in the USA [14] and in the UK [13]. Furthermore, researchers [15] conclude that although nationally representative data of prevalence rates among children in the USA were not significantly different from 2009 to 2010, more severe forms of obesity have increased over the last 14 years. In Slovenia, twenty year trends indicate that the odds for obesity (odds ratio 3.7) is growing at higher rates than overweight (odd ratio 1.7) per year, especially among boys [12]. Trends indicating higher obesity increases in boys are also reported elsewhere [1,11,13,16]. Age adjusted BMI increased in Japanese children over a 25-year-period; 6-14-year-old boys showed an increase of 0.32 kg/m2 per 10 years, and 0.24 kg/m2 per 10 years in girls [11]. In Canada, during the period from 1986 to 1996, overweight increased among boys from 11% to 33%, and from 13% to 27% among girls while obesity increased from 2% to 10% in boys and from 2% to 9% in girls [16]. Time trends in the UK (1995–2010), based on the Health Survey of England, indicated marked increases in prevalence of boys 2–15 years from 11% to 17% while girls showed increases from 12% to 15%.

Overweight and obesity are highlighted as a major public health issue in SA [17], and there is a clear need for accurate estimates of the prevalence and severity of obesity. Continued surveillance of nutritional status, as an important component of a national strategy to prevent and control both malnutrition and chronic diseases, is recommended [2]. Cross-sectional studies [7,17-20] and results of trend analyses [2,9] are available of SA children. However, there are only a few studies reporting on the prevalence of obesity longitudinally in pre-pubertal South African children. This leaves a gap in the knowledge regarding the rate of growth of this health problem. Such studies can provide accurate estimates of the rate of change in obesity prevalence among pre-pubertal children. It can also ensure the chances of improved and accurate knowledge about weight changes in the same individual, thus providing direction for future research. The association of the adiposity rebound, which is reported to occur around the age of six, and obesity in later years, further highlight that the period between 6 and 9 years may be a critical period for obesity prevention [21]. Recent findings also indicate that obesity intervention is most successful during the pre-pubertal period [22]. This study was designed to address this knowledge gap by obtaining more information about the current rate of change in overweight and obesity in pre-pubertal children in the age range 6–9 years in the North West Province (NWP) of SA taking into consideration sex, race and school SES quintiles.

## Method

### Study design, setting and population

The research formed part of the NW-CHILD (Child-Health-Integrated-Learning and Development) longitudinal study. Measurements were made at 3-intervals between 6 and 12 years (grade 1, grade 4, grade 7). This study was conducted in 1 of the 9 provinces in SA, the North-West Province (NWP) where approximately 8.2% of the national population lives. The NWP is characterised by high poverty levels especially in rural areas, unequal distribution of income between different population groups, and unemployment [23]. Income per capita in the NWP is seventh from nine provinces and it is estimated that 72.9% of children living in the NWP suffer from poverty [24].

### Sample size

The total group that were measured during baseline in 2010, when the participants were in grade 1, consisted of 816 learners (419 boys and 397 girls, 567 black, 218 white, 20 mixed ancestry and 11 Indian children), with a mean age of 6.78 years. Three years later, in 2013, 574 children with a mean age 9.87 (±038), were available for the 1^st^ interval point measurements, which represented an attrition rate of 30.1% of the original sample. Boys (n = 282, 49.12%) and girls (n = 292, 50.87) were equally distributed in the group. 27 mixed ancestry and Indian children were part of the group but were omitted from the racial comparison because of the small numbers. More black (n = 407) than white (n = 143) children were part of the group, while the number of children in the different school quintiles ranged between 96 and 130. The distribution of the learners in the different school quintiles was as follows: Quintile (Q) 1 (n = 120); Quintile 2 (n = 96), Quintile 3 (n = 130), Quintile 4 (n = 108) and Quintile 5 (n = 120). A possible bias as a result of lost subjects during follow-up were analysed using independent t-testing, calculating differences between lost subjects and those who remained in the study in 2013, in baseline height (p = 0.553, d = 0.04), mass (p = 0.03, d = 0.16), BMI (p = 0.008, d = 0.19) and fat percentage (p = 0.223, d = 0.09). No evidence of bias could be found based on the insignificant Cohen’s d-values [25].

### Sampling method

The participants were selected by means of a stratified random sample. Stratification was done by school district, gender and school quintile (Q) in collaboration with the Statistical Consultation Service of the North-West University (NWU). To determine the sample, a list of schools in the NWP was obtained from the Department of Basic Education. From the list of schools in the NWP, which are grouped in 8 education districts, each representing 12–22 regions with approximately 20 schools (minimum 12, maximum 47) per region, stratified random sampling was used to select regions and schools with regard to population density and school status (Quintile 1, i.e. schools from very poor economic sectors to Quintile 5, i.e. schools from very good economic sectors). The quintile status of a school is determined by the National Treasury, according to the National Poverty Table, obtained from the National Census data which include income, dependant ratios and levels of literacy. This poverty classification is used by the Department of Basic Education in each province to classify schools in different quintiles. Quintile 1 and 2 schools are the poorest schools and are released from paying any school fees [23]. Throughout the paper Q1-3 schools will by definition represent schools from low SES, while Q4-5 schools will represent high SES.

### Anthropometry

The anthropometric measurements included the following: height (cm), body mass (kg), skinfolds (sub-scapular and triceps, mm) and waist circumference (cm). These variables were measured by trained postgraduate students in Human Movement Sciences. All measurements were done in accordance to the protocol of the International Society for the Advancement of Kinanthropometry [26]. Height was measured barefoot to the nearest 0.1 cm by means of a Harpenden portable stadiometer (Holtain Limited, U.K.). Body mass was measured with an electronic scale (BF 511, Omron) to the nearest 0.1 kg. From the height and body mass measurements the body mass index (BMI) was calculated for each participant (body mass (kg)/height (m)^2^). The triceps and sub-scapular skinfolds were measured with a pair of Harpenden skinfold callipers and each skinfold was measured twice to obtain an average of the two measurements. [27]. Cut-off values for the sum of the triceps and subscapular skinfolds for 6 to 7-year-old overweight boys are 16–17, for overweight girls 19–22, for 6 to 7-year-old obese boys 20–24 and for obese girls 27–28. Cut-off values for 9 to 10-year-old overweight boys are 23–24, for overweight girls 29–32, for 9 to 10-year-old obese boys 34–33 and for obese girls 41–43 [27]. These skinfold measurements were selected because they show the highest relation with the overall percentage of fat in the bodies of children [28]. Intra-rater reliability was determined by intra-class correlation coefficients which showed good reliability for the subscapular (.994) and the triceps (.995) skinfolds.

The prevalence of overweight (OW) and obesity (OB) was determined by using the International age-adjusted cut-off points provided by Cole *et al*. (2000) [29]. Children have a risk for overweight and obesity if their BMI is respectively between the 85^th^ and 95^th^ percentile for age and gender. The following international cut-off values were used. It is calculated for overweight and obesity by sex between 2 and 18 years, defined as body mass index between 25 and 30 kg/m2 up to the age of 18. These cut-off values for girls are: 6 years: 17.34/19.65, 6.5 years: 17.53/20.08, 7 years: 17.75/20.51, 7.5 years: 18.03/21.01, 8 years: 18.35/21.57, 8.5 years: 18.69/22.18, 9 years: 19.07/22.81, 9.5 years: 19.45/23.46, 10 years 19.86/24.11, 10.5 years: 20.29/24.77. The cut-off values for boys are: Boys 6 years: 17.55/19.78, 6.5 years: 17.71/20.23, 7 years: 17.92/20.63, 7.5 years: 18.16/21.09, 8 years 18.44/21.60, 8.5 years: 18.76/22.17, 9 years: 19.10/22.77, 9.5 years: 19.46/23.39, 10 years: 19.84/24.00, 10.5 years: 20.20/24.57. Throughout the paper, subjects described as obese are by definition also classified as overweight.

### Ethical clearance and administrative clearance

Ethical approval for the study was obtained from the Ethics Committee of the NWU (No. 00070 09 A1). Permission was also obtained from the Department of Basic Education of the NWP and the principals from the selected schools. Informed consent had to be provided for each child by their parents or legal guardian, before they were allowed to participate in the study.

### Statistical analysis

Data was descriptively analysed by means and percentages. Linear mixed models in SPSS (version 22) were used with school as subject and an unstructured co-variance matrix to determine the main effects of race, gender and SES as well as all interaction effects, using the 2010 baseline measurements as co-variants. Frequency tables were used to determine prevalence of overweight and obesity by group, sex, race and school quintile. Significance of differences in BMI categories and shifts over time in this relationship (p < 0.05) were determined by 2-Way summary tables. Pearson Chi-square analysis determined statistical significance of differences in BMI status and relationships over time, while the Cramer’s V was used to establish effect sizes. The following values are used as an estimation of practical significance (Cramer’s V = 0.1 (small), 0.3 (moderate), 0.5 (large) [25].

## Results

Table 1 provides the statistics of obesity prevalence in 2010 and 2013, by group, but also according to sex, white and black children and different school quintiles. During follow-up in 2013, the group had a combined overweight (9.4%) and obesity (7.3%) prevalence of 16.7%, compared to that found in 2010 (Combined OW/OB = 12.7%; 8.2% OW; 4.5% OB). Prevalence during follow-up was 14.9% and 18.5% respectively for boys and girls, compared to that found during baseline (10.6% and 14.7%). White children displayed a prevalence of 27.3% compared to 13.3% in black children during follow-up, where the prevalence increased respectively from 20.3% in white children and 10.3% in black children during baseline. The change in prevalence in the different school types was much lower in Q1–Q3 schools which had only black learners compared to that found in Q4–Q5 schools which had black and white learners (Q1-9.2%–10.0%; Q2-8.3%–8.5; Q3-3.9%–7.7%, Q4-19.4%–25.9; Q5-23.3%–31.7%).

**Table 1:** **Obesity prevalence in 2010 and 2013, by group, sex, race and school quintile**

		**Normal weight**		**Overweight**		**Obese**		**Overweight & obese**	
	**N**	**2010 % (n)**	**2013 % (n)**	**2010 % (n)**	**2013 % (n)**	**2010 % (n)**	**2013 % (n)**	**2010 % (n)**	**2013 % (n)**
**Group**	574	87.3 (501)	83.3 (478)	8.2 (47)	9.4 (54)	4.5 (26)	7.3 (42)	12.7	16.7
**Boys**	282	89.4 (252)	85.1 (240)	6.7 (19)	7.8 (22)	3.9 (11)	7.1 (20)	10.6	14.89
**Girls**	292	85.3 (249)	81.5 (238)	9.6 (28)	11.0 (32)	5.1 (15)	7.5 (22)	14.7	18.5
**White**	143	79.8 (114)	72.7 (104)	13.3 (19)	16.1 (23)	6.99 (10)	11.2 (16)	20.3	27.3
**Black**	407	89.7 (365)	86.7 (353)	6.6 (27)	7.6 (31)	3.7 (15)	5.7 (23)	10.3	13.3
**Quintile 1**	120	90.8 (109)	90.0 (108)	5.8 (7)	6.7 (8)	3.3 (4)	3.3 (4)	9.2	10.0
**Quintile 2**	96	91.7 (88)	91.7 (88)	4.2 (4)	3.1 (3)	4.2 (4)	5.2 (5)	8.3	8.5
**Quintile 3**	130	96.1 (125)	92.3 (120)	2.3 (3)	3.9 (5)	1.5 (2)	3.9 (5)	3.9	7.7
**Quintile 4**	108	80.6 (87)	74.1 (80)	11.1 (12)	11.1 (12)	8.3 (9)	14.8 (16)	19.4	25.9
White	53	86.8 (46)	81.1 (43)	7.6 (4)	5.7 (3)	5.7 (3)	13.2 (7)	13.3	18.9
Black	49	73.5 (36)	65.3 (5)	14.3 (7)	18.4 (9)	12.2 (6)	16.3 (8)	26.5	34.7
**Quintile 5**	120	76.7 (92)	68.3 (82)	17.5 (21)	21.7 (26)	5.8 (7)	10.0 (12)	23.3	31.7
White	90	75.6 (68)	67.78 (61)	16.7 (15)	22.2 (20)	7.8 (7)	10.0 (9)	24.5	32.2
Black	29	79.3 (23)	68.9 (20)	20.7 (6)	20.7 (6)	0 (0)	10.3 (3)	20.7	31.0

Note- Q1–Q3 schools included only black children.

The combined OW/OB prevalence of 16.7% during follow-up, showed an increase of 4.0% over the 3-year-period in the group, increasing from 12.7% during baseline. Overweight increased with 1.2% from 8.2% to 9.4%, compared to obesity which increased more with 2.8% from 4.5% to 7.3%. Obesity prevalence also increased more in boys (3.2%) from 3.9% to 7.1% compared to that of girls (2.4%) changing from 5.2% to 7.5%. Obesity increased significantly in black (1.96%) and in white participants (4.2%). The highest rates of obesity increases were found in Q3 (1.5% to 3.9%), Q4 (8.3% to 14.8%) and Q5 (5.8% to 10.0%) schools.

Table 2 displays percentage shifts in BMI categories over the 3-year period. Overall, 87.8% of the group stayed within the BMI classification that they were classified in during the baseline measurements. A significant upward transition of 9.2% (Cramer’s V = .530) was however, found in the group, where 53 participants moved from a normal weight to overweight or obese classification based on BMI. A reverse trend was however, also observed, but among a much lower percentage of the group (n = 17, 3.0%). Similar, but bigger upward compared to downward shifts were observed in both sexes (Table 2), while more girls showed decreasing tendencies. In white children, a significant shift (Cramer’s V = .531) towards overweight and obesity took place among 14.7% (n = 21) of the group, compared to 4.9% (n = 7), shifting from overweight and obese to normal weight. Among black children, a significant upward shift towards an unhealthy BMI occurred in 7.4% children (n = 30) compared to a reverse tendency of 2.5% in 10 children (Cramer’s V = .531). This indicates that for every 3 children that move into an overweight or obese category, one transferred back to a healthier classification. The difference between increasing and decreasing tendencies in the different school quintiles was much bigger in Q3–Q5 schools (5.3%, 11.1% and 12.5% respectively) compared to in Q1 to Q3 schools (0.8% and 1.0%), where the percentages of participants showing upward or downward transitions were more or less the same. A clear upward transition to more overweight or obese categories, is however evident from Table 2, showing that children attending school quintiles associated with higher SES are more prone to become overweight or obese than children attending school quintiles associated with lowers SES.

**Table 2:** **Percentage shift in BMI categories over a 3-yr-period**

	**Group % (n)**	**Boys % (n)**	**Girls % (n)**	**White % (n)**	**Black % (n)**	**Q1 % (n)**	**Q2 % (n)**	**Q3 % (n)**	**Q4 % (n)**	**Q5 % (n)**
**Increase**	9.2 (53)	9.2 (26)	9.2 (27)	14.7 (21)	7.4 (31)	3.3 (4)	4.2 (4)	6.2 (8)	13.9 (15)	18.3 (22)
**Decrease**	3.0 (17)	2.1 (6)	7.3 (11)	5.0 (7)	2.5 (10)	2.5 (3)	3.1 (3)	0.8 (1)	2.8 (3)	5.8 (7)

% = percentage; n = number of participants in parentheses; W = White; B = Black; Q = Quintile.

Tables 3 and 4 provide descriptive data of the group (N = 574) during the baseline and follow-up measurements, according to gender, race and school quintile by means of mixed models. Descriptive values of height, body mass, BMI and fat percentage are displayed as well as the significance of main effects for follow-up measurements. Linear mixed models on baseline measurements (2010) indicated a quintile*sex interaction for height where boys in Q4 schools where practically significantly taller than girls (d = 0.82). A race*quintile interaction for BMI also indicated that black children in Q4 schools had significantly higher BMI values than white children (d = 0.5). For baseline fat percentage, both quintile and race were statistically significant, while for baseline mass only quintile had a significant effect. In the follow-up, baseline measurements of 2010 (height, mass, BMI and fat percentage respectively) were also included as co-variates to determine the effects of race, sex and quintile in the linear model. Interaction effects that showed significance in these analyses were Quintile*race for BMI and mass*quintile. The main effects of SES (school types expressed as quintiles), were significant for all variables, while sex and race were only significant with regards to fat percentage. Quintile 1 to 3 schools included only black children, but the interaction between race and quintile was evident in Q4 schools where the BMI and mass of black children were significantly higher in 2013 compared to those of white children (BMI 19.8 vs 17.6, d = 0.61 and mass 38.3 kg vs 33.9 kg,d = 0.53).

**Table 3:** **Body composition characteristics of the group and by sex and race in 2010 and 2013 and significance of differences during follow-up according to mixed models with school included as subjects**

	**Group (N = 574)**		**Boys (n = 282)**		**Girls (n = 292)**		**Sex p value**	**White (n = 143)**		**Black (n = 407)**		**Race p value**	**Mixed models MSE**
	**2010**	**2013**	**2010**	**2013**	**2010**	**2013**	**2013**	**2010**	**2013**	**2010**	**2013**	**2013**	**2013**
Height (cm)	119.76	135.37	121.3	136.0	119.9	136.0	0.588	123.7	138.0	119.3	135.3	0.723	80.9
Mass (kg)	22.59	32.29	23.3	32.9	22.9	33.7	0.662	24.8	35.5	22.5	32.4	0.240	68.3
BMI kg/^m^	15.65	17.39	15.8	17.5	15.8	18.0	0.225	16.1	18.2	15.7	17.5	0.073	13.2
Fat %	15.87	20.33	16.6	20.3	16.2	22.0	0.032	16.9	21.7	16.2	21.0	0.008	61.5

BMI = Body Mass Index; Fat % = Fat percentage; N = Participants; Significant difference, p < 0.05, MSE = mean square error.

**Table 4:** **Body composition characteristics by school quintile in 2010 and 2013 and significance of differences during follow-up according to mixed models with school included as subjects**

	**Quintile 1 (n = 120)**		**Quintile 2 (n = 96)**		**Quintile 3 (n = 130)**		**Quintile 4 (n = 108)**		**Quintile 5 (n = 120)**		**P value**	**Mixed Models MSE**
	**2010**	**2013**	**2010**	**2013**	**2010**	**2013**	**2010**	**2013**	**2010**	**2013**	**2013**	**2013**
Height (cm)	118.1	113.4	116.7	132.7	117.1	133.4	122.9	137.2	123.2	139.3	<0.001	80.9
Mass (kg)	21.4	30.3	21.0	29.1	20.5	28.9	24.7	36.1	24.7	36.4	<0.001	68.3
BMI (kg/^m^	15.4	16.7	15.3	16.4	15.0	16.3	16.2	18.7	16.2	18.6	<0.001	13.2
Fat %	14.4	18.9	15.4	18.3	14.1	17.9	17.8	23.2	17.6	23.4	<0.001	61.5

N = number of participants, BMI = Body Mass Index; Fat % = Fat percentage; Significant difference p < 0.05, MSE = mean square error.

## Discussion

The aim of this study was to determine the rate of increase in prevalence of overweight and obesity over a 3-year-period in pre-pubertal South African children. This is a first study in SA, a developing country, to provide prevalence statistics of childhood obesity obtained by follow-up measurements in pre-pubertal children, aged 6 to 9-years. 12.7% of the group were OW or OB in 2010, compared to 16.7% of the same group in 2013. The rate of increase in the group was 4.0% over the 3-year period. Increases in the group were similar in boys and girls, while white children had much higher increases compared to black children as a group, and higher SES (which included white and black children) also contributed to higher prevalences and rates of increase. A different picture of the extent of the problem emerged, when interactions of race and SES were considered, than when the participants were analysed as a group. White children had a prevalence increase of 7.0% (20.3% to 27.3%), which was double compared to black children where the increase was 3.0% (10.3% to 13.3%). However, in Q4 and Q5 schools black children showed higher increases in combined OW/OB compared to white children (8.2% vs 5.2% Q4, 9.4% vs 7.7% Q5, Table 1), which was also much higher than the increase for black children when analysed as a group (3%) or for Q1–Q3 schools were only black children were enrolled in these schools. Overall Q4 and Q5 schools which represent children from more affluent families and environments showed much bigger increases in prevalence compared to Q1 to Q3 schools which can be ascribed to improved living conditions. The main effects of SES in 2013 were significant for all variables, while sex and race were only significant for fat percentage. The Quintile*race interaction effect for BMI showed significantly higher BMI values among black children compared to white children in Q4 schools. The higher BMI of black children compared to white children in higher SES, can in part be ascribed to the economic transition of black families in South Africa, although a longitudinal study in America over a period of 17-years of racial differences [30] in 5–14 year old children, also confirmed contrasting patterns of increase in BMI between white and black children. Annual increases in BMI varied in this study from 0.60 kg/^2^ per year in white girls to 0.78 kg/^2^ per year in black girls and yearly increases in BMI before age 18 were 25% to 55% higher in black compared to white girls. Our results, however confirm the conclusions made by Rossouw et al. [8] indicating that the prevalence of childhood obesity in SA does not give a true reflection of the problem, as overweight and obesity differ markedly between age groups, boys and girls, ethnic groups and geographical areas. However, evidence of an overweight/obesity transition in school-aged children in Sub-Saharan Africa is substantiated by research [4], while the fastest growth rates of obesity among pre-school children are also found in Africa where the number of overweight and obese children in 2010, were more than double that found in 1990 [6].

In order to offer a perspective on the rate of increase of 4.0% in our group over a 3-year period, we compared this percentage increase with estimated increases that are reported by other studies. There is however, a lack of longitudinal studies, to enable direct comparisons with. Studies that were used are thus not necessarily based on the same age groups or time periods and findings obtained by trend analysis, were also incorporated. Researchers studied 450 obesity surveys of 144 countries to quantify the worldwide prevalence of OW and OB among pre-school children in Africa [6], and reported the estimated prevalence of childhood OW and OB in Africa, in 2010, as 8.5%, which they expect to reach 12.7% in 2020, indicating a predicted increase of 4.2% over a ten-year-period. A longitudinal study of 306 black children from low income families in Jamaica, reported that overweight increased by 6% (3.5% to 9.5%), from 7–8 years to 11–12%, while tracking of BMI was also high during follow-up [31]. An increase is also reported in OW and OB in first grade children in Chile [32], which is also a developing country, from 6.5% to 7.8% in boys and girls respectively in 1987, to 17% and 18.6% in 2000, which shows an increase of 12% over a 13-year period. Prevalence for developing countries is reported to have changed from 1980 to 2013 in child and adolescent boys and girls, from 8.1% to 12.9% in boys and from 8.4% to 13.4% in girls, indicating an estimate increase of 5% over this period [1].

Although not directly comparable to other studies, the rate of increase of 4.0% that were found among our 6–9-year-old group of SA children, thus displayed a more rapid increase over a shorter period of time, providing evidence of an expanding epidemic in pre-pubertal children in our study. In Japan [11], the largest odds ratio was also observed in the 6–8year-old children in whom the prevalence of obesity more than doubled from 4.2% (1976–1980) to 9.7% (1996–2000). The increase in our group was however influenced by SES and the interaction between SES and race, indicating that white children as a group and children from higher SES which included white and black children showed the most rapid increases. Statistics reported on this prevalence among white children in other SA studies [2,7,33] are also consistent with the higher prevalence found among white children. Differences reported in another SA study between ethnic groups also indicated that the results may be confounded by differences in SES [7]. However, the higher prevalence among white children compared to black children as a group, still differs from the statistics reported in developed countries such as the USA, Canada and Norway [34].

The rate of increase in overweight showed a slighter upward trend, compared to obesity prevalence which increased nearly twofold during the 3-year period. A 20 year trend analysis in Slovenia also reported a higher increase in obesity compared to overweight [12]. A trend analysis on 1–9 year old South African children, furthermore found that overweight decreased over time, while obesity increased [9]. The prevalence of obesity and severe obesity were studied over a period of 14 years in the USA in children, ages 2 to 19 years [15]. From 2011 to 2012, 17.3% of the children were obese, 5.9% met criteria for class 2 obesity (BMI ≥ 120% of the 95^th^ percentile and 2.1% met criteria for class 3 obesity (BMI ≥ 140% of the 95^th^ percentile). The researchers concluded that these rates were not significantly different from 2009 to 2010, but that more severe forms of obesity have increased over the last 14 years. Although percentages of Class 2 and 3 obesity were not determined in our study, obesity prevalence was the most severe form of OB in our study, and increased more during the 3-year-period than OW, which seems to follow the same pattern as described in the USA, a developed country.

The prevalence of obesity increased more in boys compared to girls over the 3-year period although girls still had a higher prevalence of obesity during follow-up. In addition more girls moved back to a healthier BMI compared to boys over the 3-year period. This trend of a higher increase in boys is consistent with other studies worldwide. In Canadian children, an increase is reported for OW (11 to 33% in boys, 13 to 27% in girls) and OB (2% to 10% in boys and 2 to 9% in girls) between 1981 and 1996 [16]. In 6-14-year old Japanese children, age adjusted BMI increased with 0.32 kg/^m^ per 10 years in boys and 0.24 kg/^m^ per 10 years in girls over 25-years, 6–14 years as derived from a national nutrition survey [11]. Time trends in the UK (1995–2010) based on the Health Survey of England (HSE) indicate an increase in prevalence of boys 2–15 from 11% to 17% while prevalence in girls increased from 12% to 15%. Trend analysis in the USA [14] of two large representative federal health surveys and data systems show a four-fold increase in obesity prevalence among 6-17-year-old male (5.5% to 21.6%) and a three-fold increase among female children (5.8% to 17.7%) between 1976 and 2008. The average annual rate of increase in obesity prevalence was furthermore 4.5% for male children and 3.8% for females in this study.

Prevalence in both white (4.2%) and black children (2.0%) increased significantly over the 3-year period, although white children displayed a much bigger increase and had almost double the prevalence of obesity (27.3%) than black children (13.3%) during follow-up. The combined OW/OB prevalence increase in white children was also bigger (20.3% to 27.3%) over the 3-year period than among black children (10.3% to 13.3%), although not in Q4 and Q5 schools where the increases among black children in Q4 schools were 8.2% (26.5%–34.7%) and 10.3% (20.7%–31.0%) in Q5 schools compared to those of white children (Q4, 5.6% (13.3%–18.9% and 7.7% (Q5, 24.5%–32.2%, Table 1). It further seems that South African children from higher SES have the highest prevalence and rates of increase in OW and OB. White children were all enrolled into the Q4 and Q5 schools that were part of the study, and these school quintiles represent more affluent schools, families and environments and also showed the highest combined prevalence. This differs from the findings of studies conducted in other countries such as the USA and UK, indicating the highest rates of obesity and severe obesity among children from minority groups or who are underserved by the health care system [34,35]. These studies are however conducted in developed countries while SA is considered a developing country in transition with high socio-economic disparities. It can thus be deduced that higher SES is currently associated with higher increases in overall prevalence in predominantly pre-pubertal white but also among black children in economic transition (Q4-19.4%–25.9%; Q5-23.3%–31.7%) as the increase in Q1–Q3 schools (Q1-9.1%–10%; Q2-8.3%–8.5%, Q3-3.9%–7.7%), based mainly on statistics of black children, were much less over the same period. Q1–Q3 schools enrol children from areas with high levels of food insecurity, [24,36,37], thus levels of underweight might be high in these schools. Black children in Q4 schools had significantly higher BMI and mass during follow-up compared to white children in these schools and their combined OW/OB prevalence increases were also higher. From this, the conclusion can be drawn that it can quite probably be the result of westernization and urbanization of more affluent black families. The high prevalence found among white children might also still be a result of the post-apartheid regime which exposed these children to circumstances equal to those in developed countries, such as sedentary lifestyles. Although many interrelated behaviour patterns can be contributing factors, decreased physical activity levels, among black girls, and higher food security which can contribute to higher availability and intake of processed foods, can be offered as reasons for these major differences between black children in low and high SES schools. Cultural beliefs regarding ideal body mass might be furthermore a possible contributing factor to the black and white differences that were found [36]. Spending money at school tuck shops on unhealthy foods might also play a role in the increased prevalence found in children attending Q4 and Q5 schools [38]. The prevalence of obesity that was established among 6–9-year old children by means of the first South African National Health and Nutrition Examination Survey (SANHANES-1), compared with the NFCS-2005, indicates a prevalence of 11.8% (OW 8.4% OB 3.4, mean BMI 16.2) (2013) and 10.3% (OW 7.8% OB 2.24, 2005, mean BMI 16.0) respectively [39]. This survey, however, has shortcomings in the sense that it essentially refers to African and coloured children residing in SA. When compared to our prevalence of 10.3% that were established in 2010 for 6-year-old black children, and 13.3% for 9-year old black children in 2013, a similar prevalence is confirmed in this ethnic group (11.8%). A longitudinal study of 306 black children from low income families in Jamaica, also reported low prevalence’s with an increase from baseline at 7–8 years (3.5%) to 9.5% at 11–12% [31].

Lastly, our results established that although a considerable percentage of the group transitioned over the 3-year period to an unhealthier BMI classification, a small percentage also moved to a healthier BMI. Boys and girls showed similar transition tendencies, while white children and children in schools representing higher SES (which included black children in Q4 and Q5 schools) showed higher shifts towards more unhealthy BMI’s in comparison to children in lower SES school types. Decreasing tendencies were also observed in BMI levels, although to a much smaller extent compared to the increasing tendencies that were found, resulting in a significant increase in combined OW/OB between 6 and 9 years. Although our changes in BMI were over a shorter follow-up period of 3 years, it agrees with a longitudinal study on tracking of BMI in Chinese children which was done on children who were aged between 6 and 13 years at baseline, reporting that over a 6-year period (1991–1997), that BMI remained unchanged in 40% of the group, while 30% moved to a lower or higher quintile and that overweight children were 2.8 times as likely as other children to become overweight adolescents [40]. This study, however, includes a high percentage of underweight children, and the researchers found that a smaller proportion of Chinese children in a rapidly changing society, continue to be overweight than what is reported in higher income countries.

Our study had limitations that need to be taken into consideration. This was not a nationally representative study but based on regional data of only 1 of the 9 provinces in South Africa. Research incorporating prevalence’s of all the provinces in SA are thus recommended. The strong points of the study are, however the stratified and longitudinal design, and the fact that the findings are based on real measurements and not self-reported height and weight data. This is also an on-going study with follow-up measurements due in 2016, which will provide an even more accurate picture of this growing problem among children over a period of 6 years as they move from early childhood into adolescence.

## Conclusion

These results confirm that pre-pubertal children living in SA, a developing country, are not excluded from the rising epidemic of childhood obesity with clear tracking tendencies. These young children are especially vulnerable to the side-effects associated with obesity such as adverse health risks, and developmental shortcomings because of their young age and consequently earlier exposure to unhealthy lifestyles and chronic conditions [20,36]. White learners attending schools in higher socio-economic areas (Quintile 4 and 5), showed double the increase in prevalence than black children in lower SES, although OW and especially OB were also prevalent among black learners who mostly attended schools representing lower socio-economic circumstances. Black children who attended school types associated with higher SES, showed high OW/OB prevalence and clear signs of economic transition, which impacted negatively on their body composition. This trend among black families with increasing economic opportunities is a definite concern that might need a shift in how public health nutrition and medical resources are allocated in the future. The results of this study can thus help health professionals, policy makers and experts in the field of child development to plan future preventative strategies for these children who are undergoing vast changes in diet and physical activity behaviour which include clinical management or public health intervention programmes for altering body composition levels. Awareness and educational campaigns that raise concern among parents regarding the future health problems that children might encounter due to unhealthy weight status at a young age, are also important. Culturally appropriate campaigns and strategies for interventions that would be effective for each group are also recommended. Future research, including national epidemiological and tracking studies, and intervention studies in this area is recommended to obtain a better understanding of this rising global health challenge among transitional populations. Clearly, children in developing countries, are confronted with additional challenges to their well-being, and are clearly not excluded from the dangers of lifestyle changes.

## Abbreviations

NWP: North West Province
SA: South Africa
HSE: Health Survey of England
NW-CHILD: Child-Health-Integrated-Learning and Development
NWU: North-West University
BMI: Body mass index
SES: Socio Economic Status
NSCH: National surveys of 10–17 year-old children’s health
NHANES: 1976–2008 National health and nutrition examination survey
OW: Overweight
OB: Obese
SANHANES-1: South African National Health and Nutrition Examination Survey

## References

[1] Ng M, Fleming T, Robinson M, Thomson B, Graetz N, Margono C, Mullany EC, Biryukov S, Abbafati C, Abera SF, Abraham JP, Abu-Rmeileh NME, AlBuhairan FS, Alemu ZA, Alfonso R, Ali MK, Ali R, Guzman NA, Ammar W, Anwari P, Banerjee A, Barquera S, Basu S, Bennett DA, Bhutta Z, Blore J, Cabral N, Nonato IC, Chang JC, Chowdhury R, *et al.*: Global, regional, and national prevalence of overweight and obesity in children and adults during 1980–2013: a systematic analysis for the Global Burden of Disease Study 2013*.* Lancet 2014, doi:10.1016/S0140-6736(14)60460-8. 10.1016/S0140-6736(14)60460-8 PMC4624264 24880830

[2] Reddy P, Resnicow K, James S, Kambaran N, Omardien R (2008). Underweight, overweight and obesity among South African adolescents: results of the 2002 National Youth Risk Behaviour Survey. Public Health Nutr.

[3] World Health Organisation: Obesity and Overweight Fact sheet N 311. 2013 [http://www.who.int/mediacentre/factsheets/fs311/en/]

[4] Muthuri SK, Francis CE, Wachira LJM, LeBlanc AG, Samson M, Onywera VO, Tremblay MS (2014). Evidence of an overweight/obesity transition among school aged children and youth in Sub Saharan Africa: a systematic review. PLoS One.

[5] Flynn MAT, McNeil DA, Maloff B, Mutasingwa DM, Wu M, Ford C, Tough SC (2006). Reducing obesity and related chronic disease risk in children and youth: a synthesis of evidence with ‘best practice’ recommendations. Obes Rev.

[6] De Onis M, Blossner M, Borghi E (2010). Global prevalence and trends of obesity among preschool children. Am J Clin Nutr.

[7] Armstrong MEG, Lambert MI, Sharwood KA, Lambert EV (2006). Obesity and overweight in South African primary school children – the health of the nation study. S Afr Med J.

[8] Rossouw HA, Grant CC, Viljoen M (2012). Overweight and obesity in children and adolescents: the South African problem. S Afr J Sci.

[9] Kruger HS, Steyn NP, Swart EC, Maunder EMW, Nel JH, Moeng L, Labadorios D (2011). Overweight among children decrease, but obesity prevalence remained high among women in South Africa, 1999–2005. Public Health Nutr.

[10] Padez C, Fernandes T, Mourao I, Moreira P, Rosado V (2004). Prevalence of overweight and obesity in 7–9-year-old Portuguese children: trends in body mass index from 1970–2002. Am J Hum Biol.

[11] Matsushita Y, Yoshiike N, Kaneda F, Yoshita K, Takimoto H (2004). Trends in childhood obesity in Japan over the last 25 years from the national nutrition survey. Obes Res.

[12] Kovac M, Jurak G, Kragelj LZ, Leskosek B (2014). The secular trend in the prevalence of overweight and obesity in the population of primary school children from Ljubljana (Slovenia). SJPH.

[13] United Nations Human Development Index (HDI) 2013 [http://hdr.undp.org/en/data]

[14] Singh GK, Kogan MD*:* Childhood Obesity in the United States*, 1976-2008:* Trends and current racial/ethnic, socioeconomic, and geographic disparities*. A* 75^th^ anniversary publication*.* Health Resources and Services Administration, Maternal and child health Bureau. Rockville, Maryland: *U.S. Department of Health and Human Services Administration,* 2010. http://www.mvhb.hrsa.gov/healthit/images/mchb_obesity_pub.pdf

[15] Skinner AC, Skelton JA: Prevalence and trends in obesity and severe obesity among children in the United States, 1999–2012. JAMA Pediatr 2014. Published online April 07, 2014. doi:10.1001/jamapediatrics.2014.21. 10.1001/jamapediatrics.2014.21 24710576

[16] Tremblay MS, Katzmarzyk PT, Willms JD (2002). Temporal trends in overweight and obesity in Canada, 1981–1996. Int J Obes Relat Metab Disord.

[17] Puoane T, Steyn K, Bradshaw D, Laubscher R, Fourie J, Lambert V, Mbananga N (2002). Obesity in South Africa: the South African demographic and health survey. Obes Res.

[18] Monyeki KD, Van Lenthe FJ, Steyn NP (1999). Obesity: does it occur in African children in a rural community in South Africa?. Int J Epidemiol.

[19] Jinabhai CC, Taylor M, Coutsoudis A, Tomkins AM, Sullivan KR (2001). A health and nutritional profile of rural school children in KwaZulu-Natal, South Africa. Ann Trop Paediatr.

[20] Kemp C, Pienaar AE, Schutte AE (2011). The prevalence of hypertension and the relationship with body composition in grade 1 learners in the North West province of South Africa. SAJSM.

[21] Rolland-Cachera M, Deheeger M, Bellisle F, Sempé M, Guilloud-Bataille M, Patois E (1984). Adiposity rebound in children: a simple indicator for predicting obesity. Am J Clin Nutr.

[22] Danielsson RN, Kowalski BA, Ekblom O, Marcus C (2012). Response of severely obese children and adolescents to behavioural treatment. Arc Pediatr Adolesc Med.

[23] Pauw K (2005). Profile of the North West Province: demographics, poverty, inequality and unemployment. Provided Project Background Paper.

[24] Stats SA (Statistics South Africa) (2010). Millennium Development Goals Country Report.

[25] Cohen J (1988). Statistical Power Analysis for the Behavioral Science.

[26] Marfell-Jones M, Olds T, Stewart A, Carter L (2006). International standards for anthropometric assessment. Potchefstroom, South Africa, ISAK.

[27] Lohman TG (1992). Advances in Body Composition Assessment.

[28] Meredith MD, Welk GJ (2005). Test Administration Manual: The Cooper Institute for Aerobics Research.

[29] Cole TJ, Belizzi MC, Flegal KM (2000). Establishing a standard definition for child overweight and obesity worldwide: international survey. BMJ.

[30] Freedman DS, Khan LK, Serdula MK, Dietz WH, Srinivasan SR, Berenson GS (2005). Racial differences in the tracking of childhood BMI to adulthood. Obes Res.

[31] Gaskin PS, Walker SP (2003). Obesity in a cohort of black Jamaican children as estimated by BMI and other indices of adiposity. Eur J Clin Nutr.

[32] Albala C, Vio F, Kain J, Uay R (2002). Nutrition transition in Chile: determinants and consequences. Public Health Nutr.

[33] Kruger JR, Kruger HS, McIntyre UE (2006). The determinants of overweight and obesity among 10- to 15-year-old schoolchildren in the North West Province, South Africa – the THUSA BANA (Transition and Health during Urbanisation of South Africans; BANA, children) study. Public Health Nutr.

[34] Phipps SA, Burton PS, Osberg LS, Lethbridge LN (2006). Poverty and the extent of child obesity in Canada, Norway and the United States. Obes Rev.

[35] Davison KK, Birch LL (2001). Childhood obesity: a contextual model and recommendations for future research. Obes Rev.

[36] Pienaar AE, Strydom GL: Childhood obesity: the need for practice based solutions - a South African perspective. Edited by Yuca SA. ISBN: 978-953-51-0374-5, InTech. Available from: [http://www.intechopen.com/pdfs-wm/33139.pdf]

[37] Kruger G, Pienaar AE, Coetzee D, Kruger SH (2014). Prevalence of stunting, wasting and underweight in Grade 1-learners: the NW-CHILD study. Health SA Gesondheid.

[38] Tuck shop truths study reveals poor nutritional offerings. http://www.bizcommunity.com/Article/196/329/98197.html (accessed 13 June 2013).

[39] Shisana O, Labadarios D, Rehle T, Simbayi L, Zuma K, Dhansay A, Reddy P, Parker W, Hoosain E, Naidoo P, Hongoro C, Mchiza Z, Steyn NP, Dwane N, Makoae M, Maluleke T, Ramlagan S, Zungu N, Evans MG, Jacobs L, Faber M, SANHANES-1 Team South (2013). African National Health and Nutrition Examination Survey (SANHANES-1).

[40] Wang Y, Ge K, Popkin BM (2000). Tracking of body mass index from childhood to adolescence: a 6-y follow-up study in China. Am J Clin Nutr.

